# Electrical Synapses Enhance and Accelerate Interneuron Recruitment in Response to Coincident and Sequential Excitation

**DOI:** 10.3389/fncel.2018.00156

**Published:** 2018-06-19

**Authors:** Pepe Alcami

**Affiliations:** ^1^Laboratoire de Physiologie Cérébrale, Unité Mixte de Recherche UMR8118, Université Paris Descartes and Centre National de la Recherche Scientifique, Paris, France; ^2^Laboratory of Cellular and Systemic Neurophysiology, Institute for Physiology I, Albert-Ludwigs University Freiburg, Freiburg, Germany; ^3^Grass Laboratory, Marine Biological Laboratory, Woods Hole, MA, United States; ^4^Department of Behavioural Neurobiology, Max Planck Institute for Ornithology, Seewiesen, Germany

**Keywords:** gap junction, synaptic integration, interneurons, inhibition, coincidence, cerebellum

## Abstract

Electrical synapses are ubiquitous in interneuron networks. They form intercellular pathways, allowing electrical currents to leak between coupled interneurons. I explored the impact of electrical coupling on the integration of excitatory signals and on the coincidence detection abilities of electrically-coupled cerebellar basket cells (BCs). In order to do so, I quantified the influence of electrical coupling on the rate, the probability and the latency at which BCs generate action potentials when stimulated. The long-lasting simultaneous suprathreshold depolarization of a coupled cell evoked an increase in firing rate and a shortening of action potential latency in a reference basket cell, compared to its depolarization alone. Likewise, the action potential probability of coupled cells was strongly increased when they were simultaneously stimulated with trains of short-duration near-threshold current pulses (mimicking the activation of presynaptic granule cells) at 10 Hz, and to a lesser extent at 50 Hz, an effect that was absent in non-coupled cells. Moreover, action potential probability was increased and action potential latency was shortened in response to synaptic stimulations in mice lacking the protein that forms gap junctions between BCs, connexin36, relative to wild-type (WT) controls. These results suggest that electrical synapses between BCs decrease the probability and increase the latency of stimulus-triggered action potentials, both effects being reverted upon simultaneous excitation of coupled cells. Interestingly, varying the delay at which coupled cells are stimulated revealed that the probability and the speed of action potential generation are facilitated maximally when a basket cell is stimulated shortly after a coupled cell. These findings suggest that electrically-coupled interneurons behave as coincidence and sequence detectors that dynamically regulate the latency and the strength of inhibition onto postsynaptic targets depending on the degree of input synchrony in the coupled interneuron network.

## Introduction

Inhibitory interneurons control the timing of signals carried between and within brain areas by inhibiting action potential generation in principal neurons (Pouille and Scanziani, 2001; Brunel et al., 2004; Mittmann et al., 2005; Chu et al., 2012; Blot and Barbour, 2014). The properties of inhibition are shaped by an interplay of synaptic, cellular and network mechanisms (Hu et al., 2014). Synaptic connections between interneurons play a critical role in coordinating the activity of interneuron networks, ultimately controlling the time windows during which action potentials can be generated by principal cells (Bartos et al., 2002). Interneuron networks communicate through a combination of chemical and electrical synapses (ESs), the latter being formed by intercellular channels that mediate electrical coupling (Galarreta and Hestrin, 2001a; Bennett and Zukin, 2004). Classically, ESs have been proposed to equalize the membrane potential of electrically-coupled neurons, thereby synchronizing subthreshold and spiking activity and contributing to network oscillations (Mann-Metzer and Yarom, 1999; Deans et al., 2001; Galarreta and Hestrin, 2001a; Hormuzdi et al., 2001; Kopell and Ermentrout, 2004; van Welie et al., 2016). However, recent studies have questioned a major role of ESs in synchronizing oscillatory activity and action potentials in a number of networks (Hjorth et al., 2009; Vervaeke et al., 2010; Salkoff et al., 2015). Additionally, computational models suggest that ESs may be effective in synchronizing neural networks only under certain conditions (Tchumatchenko and Clopath, 2014).

ESs have been alternatively proposed to dynamically influence the excitability of coupled networks by allowing them to detect a temporally-coincident activation of the network (Galarreta and Hestrin, 1999, 2001b; Veruki and Hartveit, 2002; Rela and Szczupak, 2004; Hjorth et al., 2009; Amsalem et al., 2016). According to this hypothesis, ESs decrease the excitability of electrically-coupled interneurons by leaking electrical current. This effect is reverted when coincident currents impinge on coupled cells, in which case the excitability of coupled neurons is comparatively increased and neurons integrate coincident inputs more efficiently (Di Garbo et al., 2007; Hjorth et al., 2009). Here, I test the influence of ESs on the rate, the probability and the latency at which coupled cerebellar interneurons are recruited in response to temporally-separated, coincident or sequential stimuli.

Two complementary approaches were implemented in mouse and rat cerebellar basket cells (BCs), which are strongly electrically-coupled in both the juvenile and the adult brain (Alcami and Marty, 2013; Kim et al., 2014; Rieubland et al., 2014). BCs exquisitely control the time-window for action potential generation from Purkinje cells (Brunel et al., 2004; Mittmann et al., 2005; Chu et al., 2012; Blot and Barbour, 2014). However, the dependence of BC action potential generation on ESs remains elusive. In the first approach, intracellular depolarizing currents were injected in electrically-coupled BCs. The electrical activity of BCs was compared when current was injected in only one BC or in two coupled BCs simultaneously, in which case the voltage difference between both sides of the gap junction is expected to be largely reduced, thereby reducing the current leakage through the ES between the two BCs and increasing their excitability. When a BC generates an action potential, it evokes postsynaptic spikelets in coupled cells, further influencing their excitability. Thus, comparing simultaneous and non-simultaneous excitation of coupled cells makes it possible to infer the contribution of ES between two BCs to their excitability in response to both coincident and non-coincident inputs. These two stimulation patterns are noteworthy in the cerebellar cortex, where granule cell axons are simultaneously activated in spatial clusters. BCs are therefore expected to be excited with high synchrony *in vivo* in the center of the spatial cluster of active granule cell axons, or non-simultaneously at its periphery (Cohen and Yarom, 1998; Cramer et al., 2013). This manipulation also overcomes major shortcomings of pharmacological manipulations to block ESs, which directly affect synaptic and intrinsic properties (Tovar et al., 2009). The second approach consisted of comparing the firing responses of BCs to evoked synaptic glutamatergic events in wild-type (WT) and in Cx36−/− mice, which lack electrical coupling between BCs (Alcami and Marty, 2013). Both approaches concur to suggest a major role for ESs in controlling interneuron recruitment.

## Materials and Methods

### Animals and Slice Preparation

Sagittal slices (200 μm thick) were prepared from the cerebellar vermis of Sprague-Dawley rats (PN 12–15), C57BL/6J WT mice or Cx36−/− mice (PN 11–13). Slices were prepared as previously described (Alcami and Marty, 2013). Rats or mice of either sex kept at 12 h light/12 h darkness cycle were decapitated before removal of the cerebellum. Cerebellar slices were made using a Leica VT 1000S vibratome while the cerebellum was bathed in an ice-cold artificial cerebrospinal fluid (composition: 130 mM NaCl, 2.5 mM KCl, 26 mM NaHCO_3_, 1.3 mM NaH_2_PO_4_, 10 mM glucose, 2 mM CaCl_2_, and 1 mM MgCl_2_; osmolarity 300 mOsm) or in an alternative ice-cold solution (composition: 87 mM NaCl, 25 mM NaHCO_3_, 2.5 mM KCl, 1.25 mM NaH_2_PO_4_, 10 mM glucose, 75 mM sucrose, 0.5 mM CaCl_2_, and 7 mM MgCl_2_), equilibrated with 95% (vol/vol) O_2_ and 5% (vol/vol) CO_2_ (pH 7.4). Slices were incubated for 40 min at 34°C in oxygenated ACSF and kept at room temperature. Experiments on mice were not blind: the animals were identified before the experiments were performed. Cx36−/− mice were kindly provided by H. Monyer, Department of Clinical Neurobiology, Heidelberg University Medical Center, Heidelberg. All experimental procedures were designed in accordance with the institutional, national and European animal care guidelines and legislations, in accordance with the European Directive 2010/63/UE, the animal care guidelines of Paris Descartes University (approval number A-750607), the X-10/18S license at Freiburg University and the Institutional Animal Care and Use Committee (IACUC) approval at MBL (13-07E).

### Electrophysiology: General Procedures

BCs were identified as small-diameter cell bodies (~10 μm) located in the internal third of the molecular layer. Recordings were performed at room temperature, ~21°C or at near-physiological temperatures, ~34°C as specified in the text, with HEKA EPC9 or EPC10 amplifiers and Patchmaster v2x32 software or with a Multiclamp700B amplifier (Axon Instruments) and a custom-made Igor-based program (FPulse, Dr. Fröbe, Institute of Physiology I, University of Freiburg^1^). Electrophysiological data were analyzed with the help of Neuromatic (a collection of Igor Pro functions for analysis of electrophysiological data^2^). Electrical coupling was detected by injecting a hyperpolarizing current pulse of 200–600 ms in one cell and recording the voltage change in the other cell. The drugs used to block chemical transmission were: SR 95531 (Tocris, 10 μM) and CNQX (Tocris, 20 μM).

### Whole-Cell Recordings

The internal recording solution contained: 144 mM K gluconate, 6 mM KCl, 4.6 mM MgCl_2_, 2 mM CaCl_2_, 1 mM EGTA, 10 mM HEPES, 0.4 mM Na_2_GTP, 4 mM Na_2_ATP; pH 7.4; osmolarity: 295 mosm. Whole-cell recording pipettes had an open tip resistance from 6 MΩ to 9 MΩ. Junction potentials were taken into account in the presentation of whole-cell recording data by subtracting 12 mV from the values read from the amplifier. Experiments on coupled cells were performed in pairs with no direct chemical connections located at intersomatic distances smaller than 50 μm.

### Extracellular Stimulation

An A-M systems isolated pulse stimulator (model 2100) was used to deliver 0.2 ms long pulses at 20–70 V. The stimulation electrode with tip resistance of 3–6 MΩ was placed in the granule cell layer, and the stimulation intensity was increased until an excitatory postsynaptic current or potential was observed. The location of the stimulation electrode was changed when no response was observed, and this procedure was repeated until a response was recorded. Excitatory postsynaptic currents (EPSCs) and EPSPs were recognized by their fast kinetics and reversal close to 0 mV, compared to slower GABAergic events reversing around −60 mV (Mejia-Gervacio et al., 2007).

### Estimate of Junctional Conductance

The conductance of gap junctions was estimated in both directions G_j(1→2)_ and G_j(2→1)_; from cell 1 to cell 2 and from cell 2 to cell 1, respectively) by the following equations (Hoge et al., 2011):

$${\text{G}}_{\text{j}(\text{1}->\text{2})}=1/[[{\text{R}}_{\text{in}(\text{cell 1})}*{\text{R}}_{\text{in}(\text{cell 2})}-{\text{R}}_{\text{t}{\left(1->2\right)}^{2}}]/{\text{R}}_{\text{t}(\text{1}->\text{2})}];$$

$${\text{G}}_{\text{j}(\text{2}->\text{1})}=1/[[{\text{R}}_{\text{in}(\text{cell 2})}*{\text{R}}_{\text{in}(\text{cell 1})}-{\text{R}}_{\text{t}{\left(2->1\right)}^{2}}]/{\text{R}}_{\text{t}(\text{2}->\text{1})}],$$

where Rincell1 and Rincell 2 are the input resistance of cells 1 and cell 2, Rt1 → 2 is given by the amplitude of the voltage response in cell 2 divided by the amplitude of the current pulse in cell 1.

### Statistical Analysis

Data are expressed as mean ± SEM. Statistical tests were performed with Igor Pro software. The tests used throughout the manuscript are: the Wilcoxon Signed-Rank (WSR) test for paired experiments, the Wilcoxon-Mann-Whitney (WMW) two sample rank test for two-sample datasets, and the linear correlation test, as specified in the text.

## Results

### ESs Mediate Coincidence Detection and Modulate BCs Firing Rate

**Figure 1:**
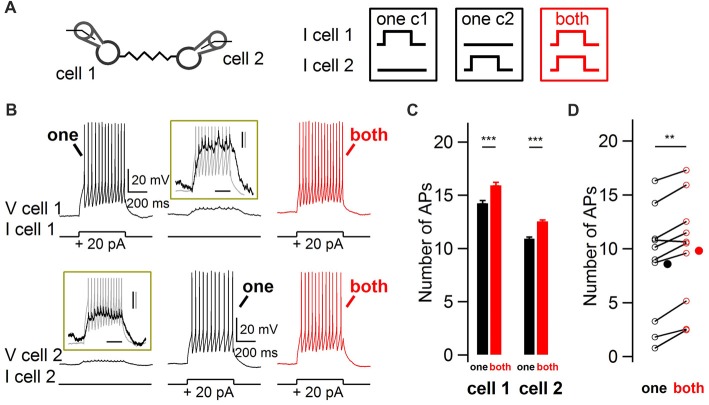
Impact of coincidence detection by electrical synapses (ESs) on basket cell (BC) firing rate. **(A)** Two electrically-coupled BCs were recorded in current-clamp mode. Five-hundred millisecond duration 20 pA current pulses were injected in one cell (“one”, black) or simultaneously in both cells (“both”, red). **(B)** Increase in BC firing rate when both BCs were simultaneously depolarized. Representative membrane potential recordings from two simultaneously-recorded electrically-coupled BCS at membrane potentials of ~−70 mV. Top traces, cell 1. Bottom traces, cell 2. Insets enlarge the subthreshold depolarizations induced by the current injection in the other cell, shown in gray. Scale bars, 200 ms, 2 mV (black) and 10 mV (gray). **(C)** Average increase (±SEM) in the number of action potentials (APs) from cells shown in **(B)** when their electrically-coupled partner was simultaneously injected with a depolarizing current pulse (Wilcoxon-Mann-Whitney (WMW) test, ***P < 0.001). **(D)** Summary results for 10 cells showing the average increase in the number of action potentials when an electrically-coupled cell was simultaneously depolarized (Wilcoxon Signed-Rank (WSR) test, **P < 0.01). Open symbols, individual cells and filled symbols, average values.

In order to explore the modulation of BCs firing rate by ESs in response to coincident vs. non-coincident excitation, paired whole-cell recordings from electrically-coupled BCs were performed in acute slices from juvenile rats. Action potential firing responses of nearby electrically-coupled BCs were evoked by 500 ms duration depolarizing current pulses, first separated in time and then simultaneously in both cells (Figure 1). The insets in Figure 1B show the subthreshold depolarization as well as the spikelets riding on top of it, evoked by a suprathreshold current injection in the other recorded cell. When both cells were simultaneously depolarized, the number of action potentials increased in both cells, from 8.6 to 9.8 action potentials in 500 ms (10 cells, one-tail WSR test, *P* = 0.002; Figure 1D). Therefore, coupled BCs detect simultaneous excitation and as a result increase their firing rate.

### The Simultaneous Depolarization of Two Coupled Cells Decreases Action Potential Latency

**Figure 2:**
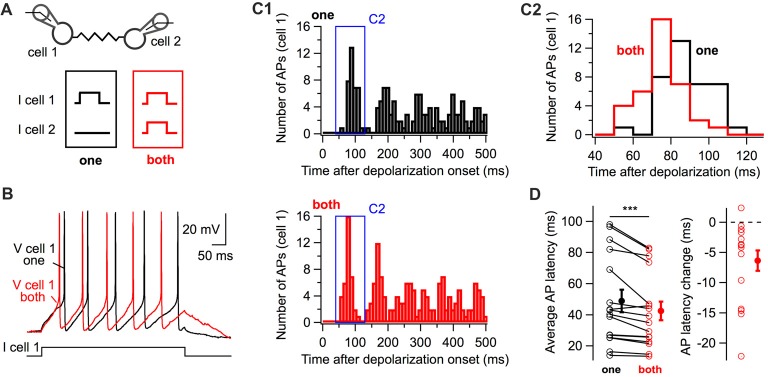
The simultaneous depolarization of a BC regulates the latency of action potential generation in an electrically-coupled BC.** (A)** Two BCs were recorded in current-clamp mode. Five-hundred millisecond 10 or 20 pA current pulses were injected in one cell (“one” in black) or in both cells (“both” in red) simultaneously. **(B)** Representative membrane potential traces from cell 1 (“one”, black, “both”, red). **(C)** Peri-stimulus time histogram (PSTH) computed from all trials for the cell in **(B)** showing the shortening of action potential latency when both cells are stimulated **(C1)**. The time-window of the PSTH contributed by the first action potential is enlarged in **(C2)**. The distribution of latencies is shifted towards lower values when both cells are simultaneously depolarized. **(D)** Summary data showing the decrease in the latency of the first action potential triggered by the positive current injection when an electrically-coupled cell is simultaneously depolarized (WSR test, ***P < 0.001). Open symbols represent individual cells and filled symbols average values ± SEM. Left, average AP latencies. Right, change in the latency of the first action potential generated (latency when both cells were depolarized subtracted by the latency when cells were individually depolarized).

The experimental paradigm shown in Figure 1 was used to investigate whether coupled interneurons generate action potentials with shorter latency upon simultaneous depolarization of a coupled cell (Figure 2A). When both cells were simultaneously depolarized with small-amplitude depolarizing currents, action potentials were generated at shorter latencies than when they were individually depolarized (42.5 ± 6.0 ms and 48.9 ± 7.2 ms respectively; 16 cells; one-tail WSR test, *P* = 0.0002; Figure 2D) as shown in representative voltage traces (Figure 2B) and peri-stimulus histograms (PSTH, Figure 2C). Thus, simultaneously depolarizing a coupled cell is sufficient to decrease the latency of the first action potential generated by a BC in response to a depolarizing current.

### Simultaneous Trains of Short-Duration Near-Threshold Pulses Increase Action Potential Firing Probability in a Frequency-Dependent Manner and Decrease Action Potential Latency

The experiments of Figures 1, 2 consist of long-lasting steady-state current injections, a regime at which one would expect electrically-coupled cells to be efficiently loaded through ESs. Indeed, electrical synaptic transmission acts as a low-pass filter and the time scale needed to load the membrane of electrically-coupled cells through ES is in the range of tens of milliseconds (Bennett and Zukin, 2004; Alcami and Marty, 2013). However, it is unclear whether electrical transmission significantly affects the integration of fast excitatory chemical events, which take place on the millisecond time scale.

**Figure 3:**
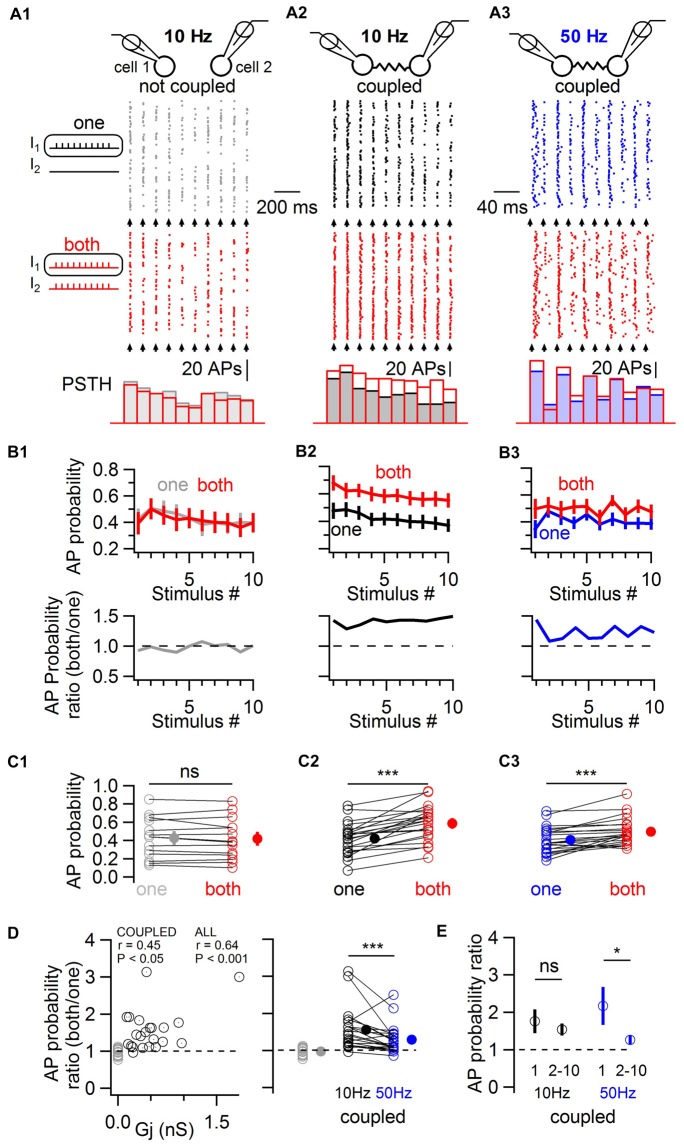
Electrically-coupled BCs detect coincident short pulses in a frequency-dependent manner. **(A)** Short current pulses of 1 ms in duration were applied individually to only one cell (“one”, gray, black and blue traces in **A1–A3**, respectively) or to both cells simultaneously (“both”, red traces in **A1–A3**). A train of ten current pulses at 10 Hz was injected in non coupled cells **(A1)**, in coupled cells **(A2)**, and at 50 Hz in coupled cells **(A3)**. Top, diagram of the recorded cells. Middle, raster plots of action potentials recorded in one of the two cells stimulated alone (top raster plot) or coincidentally with another cell (bottom raster plot). Bottom, corresponding PSTH for each condition. The timing at which current pulses were injected is indicated by arrows. **(B)** Top, summary results (mean ± SEM) showing the average action potential probability evoked by each current pulse in the train in response to individual (“one”) or simultaneous (“both”) depolarizations in 14 control cells **(B1)** and 22 coupled cells** (B2,B3)**. Bottom, ratio of average action potential probability (average action potential probability when both cells are stimulated divided by the average action potential probability when individual cells are stimulated). **(C)** Summary results comparing the average action potential probability for individual cells in response to the 10 independent and simultaneous stimuli (open symbols), and average ± SEM (filled symbols). WSR test, ns *P* > 0.05 for non-coupled cells in **(C1)**, ***P < 0.001 in **(C2,C3)**. **(D)** Left, the individual ratio of action potential probability correlates with the junctional conductance (Gj) between recorded cells. Coupled cells are represented by black circles and non-coupled cells, by gray circles. Right, average action potential probability ratios are larger in response to 10 Hz than to 50 Hz stimulation. WSR test, ***P < 0.001. **(E)** Action potential probability ratio (±SEM) in response to the first and to subsequent stimuli in the train (stimulus number 2–10) showing short term depression in the facilitation of firing in response to coincident excitation at 50 Hz (WSR test, *P < 0.05) but not at 10 Hz (WSR test, ns *P* > 0.05).

In order to induce a fast excitation of BCs mimicking physiological patterns of chemical excitatory transmission, trains of 10 near-threshold current pulses of short duration (1 ms in duration) were applied to one or to both cells simultaneously, evoking action potentials with an average action potential probability of ~0.4 (Figure 3). These experiments were performed in the presence of blockers of fast GABAergic and glutamatergic transmission to rule out any involvement of these forms of transmission to the observed phenomenon. In the absence of electrical coupling, the simultaneous near-threshold 10 Hz activation of non-coupled pairs did not evoke any increase in action potential probability relative to their individual stimulation (0.42 ± 0.06 and 0.43 ± 0.06 respectively, one-tail WSR, *P* = 0.73; 14 cells from seven pairs; Figure 3A1,B1,C1). By contrast, in electrically-coupled pairs, the average action potential probability increased from 0.42 ± 0.04 to 0.59 ± 0.04 (one-tail WSR, *P* = 10^−6^, 22 cells from 11 pairs, Figure 3A2,B2,C2). Therefore, electrical coupling mediates an increase in action potential probability when coupled cells are simultaneously excited. Increases in action potential probability across cells correlated with the conductance of the gap junction G_j_ (linear correlation test, *r* = 0.45, *P* = 0.018, Figure 3D). If non-coupled cells were included (gray circles in Figure 3D), the correlation and significance increased (*r* = 0.64, *P* = 1.4 * 10^−5^).

Spiking coupled cells interact in complex ways which are not easy to predict (Minneci et al., 2007; Hjorth et al., 2009) and inhibitory, frequency-dependent interactions of spiking cells may counteract increases in action potential probability in response to coincident stimulation (Dugué et al., 2009; Vervaeke et al., 2010; Russo et al., 2013). Therefore, the stimulation protocol was repeated in the same coupled pairs, this time at 50 Hz (Figure 3A3,B3,C3). Near-threshold stimulations at 50 Hz revealed a less pronounced increase in action potential probability when two cells were simultaneously stimulated, from 0.41 ± 0.03 to 0.50 ± 0.03 (one-tail WSR, *P* = 2 * 10^−5^). The average firing ratio, defined as the average across cells of the ratio of the action potential probability when each coupled cell was stimulated simultaneously with another cell divided by the action potential probability when it was stimulated alone, differed for both frequencies. The average ratio was 1.56 ± 0.12 for 10 Hz and 1.29 ± 0.08 for 50 Hz (one-tail WSR, *P* = 0.0006, Figure 3D). At 50 Hz, coincidence detection was maximal for the first action potential generated in response to a train of near-threshold stimulations (average action potential probability ratio = 2.17 ± 0.47), depressing for subsequent stimuli in the train (1.26 ± 0.08, one-tail WSR, *P* = 0.013, Figure 3E), a depression that was not observed at 10 Hz (1.76 ± 0.28 for the first stimulus, 1.54 ± 0.12 for the 9 following stimuli, one-tail WSR, *P* = 0.25). Coincidence detection by ESs between BCs is therefore characterized by short term dynamics (namely, short-term depression) at 50 Hz, but not at 10 Hz.

**Figure 4:**
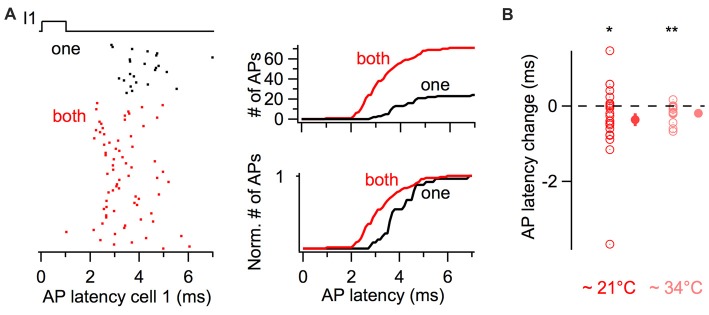
Simultaneous short-duration current pulses decrease action potential latency. **(A)** Action potential latency decrease in response to a 1 ms long current injection in a cell when a coupled cell was simultaneously stimulated (“both” in red), relative to a current injection in only the reference cell (“one” in black). Left, raster plot of action potentials showing a larger number and shorter latency of evoked action potentials. Right, cumulative histograms of action potential latency from the same cell. Top, cumulative histograms. Bottom, normalized cumulative histograms. **(B)** Summary results showing the average decrease in action potential latency when current pulses were injected in both cells at room temperature (*n* = 22 cells) and at near-physiological temperature (*n* = 14 cells). WSR test, *P < 0.05, **P < 0.01. Open symbols, individual experiments; filled symbols, mean ± SEM.

The latency of action potentials in response to the first stimulus in the train was also quantified (Figure 4). The raster plot and the cumulative histogram in Figure 4A show action potentials generated with shorter latencies by a BC when a coupled cell was simultaneously depolarized. When both cells were simultaneously excited by near-threshold short-duration current pulses, action potential latency decreased on average by 0.33 ms from 5.47 ± 0.59 ms to 5.14 ± 0.48 ms (22 cells, one-tail WSR, *P* = 0.039, Figure 4B).

Since previous experiments were performed at room temperature (~21°C) and the kinetics of membrane signaling are affected by temperature, I performed an independent set of experiments consisting of short-duration depolarizing current pulses at 10 and 50 Hz at near-physiological temperature (~34°C). The firing probability increased for simultaneous 3 ms long current injections at 10 Hz, from 0.41 ± 0.04 Hz to 0.66 ± 0.04 (one-tail WSR, *P* = 6 * 10^−5^; 14 cells from seven pairs) and to a lesser extent at 50 Hz from 0.47 ± 0.03 to 0.56 ± 0.03 (one-tail WSR, *P* = 0.002). In these pairs, the average latency of action potentials also decreased upon simultaneous depolarizations from 3.62 ± 0.13 ms to 3.43 ± 0.11 ms (one-tail WSR, *P* = 0.002, Figure 4B).

Thus at synaptically-relevant time scales, owing to their coupling through ESs, coupled cells differentially regulate their firing response to coincident and non-coincident near-threshold stimuli, the former being encoded with a higher action potential probability and a submillisecond decrease in action potential latency.

### ESs Control the Probability and the Latency of Action Potential Generation by BCs in Response to Excitatory Glutamatergic Inputs

ESs enable coincidence detection of short-duration excitatory currents (Figures 3, 4), suggesting that they may affect action potential generation in response to glutamatergic events, which typically display fast kinetics in interneurons (Jonas et al., 2004; Mejia-Gervacio et al., 2007). It has been proposed that the main effect of ESs is to decrease the occurrence of synaptically-evoked action potentials in response to uncorrelated excitatory inputs in coupled networks by a shunting effect, an effect that is minimized or reverted by coincident events (Hjorth et al., 2009). Accordingly, one would expect a net increase in action potential firing and a reduction in their latency in response to isolated synaptic inputs in BCs devoid of ESs.

**Figure 5:**
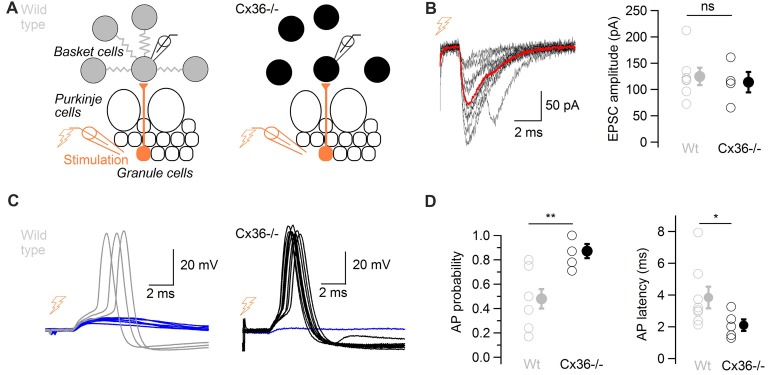
ESs control the probability and the latency of action potentials in response to glutamatergic inputs.** (A)** Diagram showing the position of the stimulation and recording electrodes. The granule cell layer was stimulated extracellularly, evoking glutamatergic synaptic events in a BC recorded in whole-cell configuration in wild type (WT; left) and in Cx36−/− mice (right). **(B)** Left, 10 evoked excitatory postsynaptic currents (EPSCs) recorded in voltage-clamp in one BC (failures not shown), average in red. Traces were aligned to EPSC onset. Right, summary data comparing recorded EPSC amplitudes in WT and Cx36−/− mice. Amplitudes did not differ (*n* = 4 Cx36−/−, *n* = 7 WT cells, WMW test, ns *P* > 0.05). Open symbols, individual experiments; filled symbols, average ± SEM. **(C)** EPSPs trigger action potentials with higher probability and shorter latency in Cx36−/− mice. Representative membrane potential recordings of a BC in response to granule cell stimulation in a WT mouse (left) and in a Cx36−/− mouse (right) at −59 ± 1 mV. Traces have been aligned to EPSP onset, 10 synaptic stimulations shown for each condition. **(D)** Summary results showing the increase in action potential probability in response to granule cell layer stimulation (left, *n* = 5 Cx36−/−, *n* = 8 WT cells, WMW test, **P < 0.01) and the decrease in action potential latency (right, WMW test, *P < 0.05) in Cx36−/− mice. Open symbols, individual experiments; filled symbols, average ± SEM.

Cx36−/− mice, which lack electrical coupling between BCs (Alcami and Marty, 2013), were used to evaluate the overall contribution of ESs to action potential generation in response to glutamatergic events. Minimal extracellular stimulations of the granule cell layer were performed in WT and in Cx36−/− mice in order to activate presynaptic granule cells (Figure 5A). Stimulations evoked glutamatergic EPSCs of comparable amplitudes in BCs from both WT and from Cx36−/− mice (124.9 ± 16.5 pA vs. 113.8 ± 19.6 pA respectively, two-tail WMW test, 7 WT cells, 4 Cx36−/− cells, *P* = 0.53; Figure 5B), suggesting that no compensation of EPSC size occurs in BCs from Cx36−/− mice. Because excitatory synaptic events occasionally evoke action potential firing in BCs (Barbour, 1993), this probability was quantified. The probability that an excitatory postsynaptic potential (EPSP) evoked an action potential at −59 ± 1 mV was higher in Cx36−/− mice than in WT mice (0.87 ± 0.06 vs. 0.48 ± 0.08 respectively; one-tail WMW test, *n* = 8 WT cells and *n* = 5 Cx36−/− cells, *P* = 0.005). EPSPs in BCs from Cx36−/− mice generated action potentials at shorter latencies than in WT mice (2.1 ± 0.4 ms vs. 3.8 ± 0.7 ms respectively; one-tail WMW test, *P* = 0.03; Figure 5C,D).

These experiments suggest that ESs decrease the probability and increase the latency at which BCs generate action potentials in response to isolated excitatory events.

### Time-Window for Enhanced Firing in Electrically-Coupled BCs

**Figure 6:**
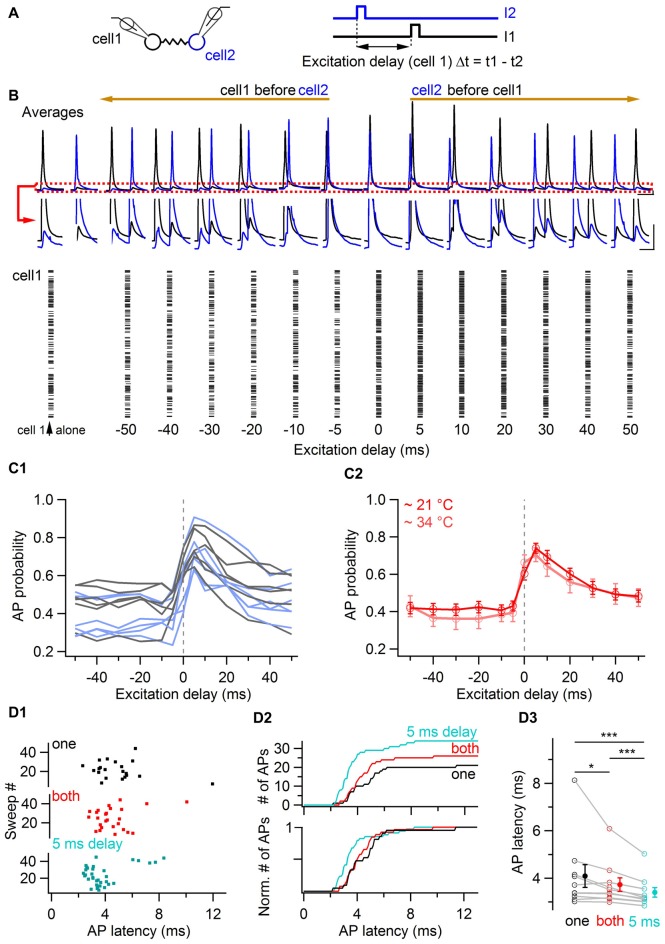
Coincidence and sequence detection by electrically-coupled BCs.** (A)** Two electrically-coupled cells were stimulated by a 3 ms duration current pulse with a delay Δt. The stimulus delay varied between −50 ms and +50 ms. **(B)** Top, average voltage traces recorded from two representative cells showing the increase of BC membrane voltage of both cells when they were excited at null and positive delays after a coupled cell. The red box is enlarged. Scale bars, 2 mV and 40 ms. Bottom, raster plot from a cell showing an increase in the number of action potentials when it is excited at null and positive delays after a coupled cell. **(C1)** Summary data representing the action potential probability of each cell as a function of the excitation delay relative to an electrically-coupled cell (gray and light blue traces corresponding to cell 1 and cell 2 from each coupled pair respectively, 12 cells from six pairs). The average action potential probability computed for all cells is shown in **(C2)**, in red at room temperature (12 cells) and in pink at near-physiological temperature (10 cells). Data represent mean ± SEM. **(D)** Action potential latency at physiological temperatures in response to independent (black), coincidental (red) and sequential excitation (turquoise). **(D1)** Raster plot for independent, simultaneous and delayed current injections in a reference cell (origin of *x*-axis is the onset of current injection in the cell). **(D2)** Cumulative histogram of action potential latency for independent (black), simultaneous (red) and delayed stimulation (turquoise) showing an increased number and a decreased latency of action potentials in response to simultaneous and delayed stimulations. **(D3)** Summary plot showing the average latency of action potentials in the three conditions. WSR test, *P < 0.05; ***P < 0.001 Open symbols, 10 individual experiments; filled symbols, mean ± SEM.

In order to investigate the time-window of relative excitation of two coupled cells at which they increase their probability of generating action potentials, the delay between short (3 ms duration) near-threshold current pulses was systematically varied in pairs of electrically-coupled BCs, evoking action potentials with an average probability of ~0.4 when individually stimulated (Figure 6). The raster plot of a representative cell is shown in Figure 6B as a function of the excitation delay Δt, defined as the difference between the time of current pulses injected in the examined cell and the time of current pulse injection in a coupled cell. This delay was varied from −50 ms to +50 ms (i.e., from the examined cell being stimulated 50 ms before a coupled cell to the examined cell being stimulated 50 ms after a coupled cell). Action potential probability increased for both cells when the stimulation delay was null and it maximally facilitated when a coupled cell was stimulated 5 ms before. Beyond 5 ms, action potential probability decreased towards baseline values for increasing values of Δt. Similar time-windows for increases in action potential probability were observed in 12 cells from six pairs (Figure 6C). The action potential probability at variable delays was normalized to the action potential probability when cells were individually stimulated (at Δt = 400 ms). The average of this ratio at Δt = 0 ms was 1.46 ± 0.12, and it peaked at a value of 1.79 ± 0.13 at Δt = 5 ms after the stimulation of a coupled cell. A similar result was found in independent experiments at near-physiological temperature, at which the maximal increase in firing also occurred for delays of Δt = 5 ms (pink trace in Figure 6C2). The average action potential probability ratio at near-physiological temperature was 1.88 ± 0.24 at Δt = 5 ms vs. 1.75 ± 0.23 at Δt = 0 ms (*n* = 10 cells). In these experiments, the latency of action potentials from a cell activated 5 ms after a coupled cell was 0.69 ms shorter than the action potential latency quantified from individual current injections in the same cells (3.40 ± 0.20 ms vs. 4.09 ± 0.48 ms, *P* < 0.001, *n* = 10 cells, one-tail WSR test) and 0.33 ms shorter than the latency observed in the same cells for simultaneous injections (3.73 ± 0.29 ms, *P* < 0.001, one-tail WSR test, Figure 6D). These results suggest that coupled BCs behave more efficiently as fast sequence detectors (~5 ms) than as coincidence detectors in terms of both their action potential probability and their action potential latency.

**Figure 7:**
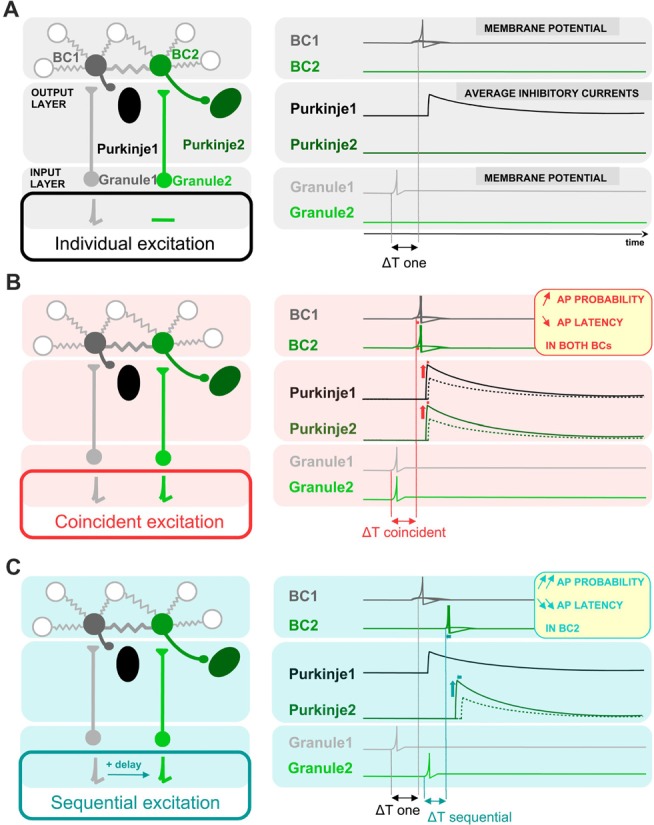
Schematic representation of the proposed impact of ESs between BCs on the average latency and amplitude of inhibition of postsynaptic Purkinje cells. Schematic drawing showing a simplified representation of the cells presynaptic and postsynaptic to BCs (left), and the activity of BCs and the inhibition of Purkinje cells in response to granule cell-mediated stimulation of BCs (right). BCs receive excitatory glutamatergic inputs from granule cells and they themselves inhibit postsynaptic Purkinje cells. Two circuits formed by one granule cell, one BC and one Purkinje cell are shown next to each other, and connected by ESs between the two BCs. Three different patterns of presynaptic granule cell activity are illustrated in **(A–C)**: independent excitation of granule cells (**A**, only granule cell 1 is stimulated), simultaneous excitation of both granule cells **(B)** and sequential excitation of granule cell 2 after granule cell 1 **(C)**. The network of coupled BCs is larger than two cells, as represented by the additional coupled gray BCs in the network. **(A)** In response to the excitation of granule cell 1, BC1 is excited with a given probability that EPSPs trigger APs, evoking an inhibitory current in its postsynaptic Purkinje cell (Purkinje cell 1) with a given average amplitude. **(B)** The simultaneous excitation of BCs by simultaneously-active granule cells evokes an increase in action potential probability and a decrease in action potential latency in BCs, and thereby an increase in the average inhibitory current received by both Purkinje cell 1 and Purkinje cell 2 and a decrease in its latency from granule excitation, relative to the inhibition that they would receive if only one BC was depolarized. Dotted line, voltage trace when cells are excited individually. **(C)** In the scenario of a sequential activation of BCs, only the inhibitory current evoked by BC2, the BC activated sequentially after its electrically-coupled BC, is changed relative to **(A)**: the action potential of BC2 takes place with an even shorter latency and higher probability than in **(B)**. In contrast, the first BC to be excited, BC1, has the same action potential probability and latency as in **(A)**, and therefore the average current evoked by the first BC to be excited does not change its amplitude nor its latency.

Results and their proposed implications are schematically represented in Figure 7. In comparison with an independent excitation of coupled cells by presynaptic granule cells (Figure 7A), the simultaneous excitation of BCs by synchronous presynaptic activity (depicted by simultaneously-firing granule cells in Figure 7B) is expected to evoke a more probable and faster action potential generation in BCs, and thereby a larger and faster average synaptic current in the postsynaptic Purkinje cells in response to granule cell activation. In the case of coupled cells being excited sequentially, the recruitment, and thereby the inhibition from the cell activated within a short delay will be further accelerated and increased (Figure 7C).

## Discussion

### ESs Enhance and Accelerate the Recruitment of Interneurons in Response to Coincident Stimuli Relative to Temporally-Separated Stimuli

ESs are ubiquitous in neural networks across phyla (Galarreta and Hestrin, 2001a; Bennett and Zukin, 2004; Rela and Szczupak, 2004). However, their impact on the recruitment of coupled neurons and on their coincidence detection abilities remains mostly overlooked. Experiments confirm that ESs control neuronal excitability by reducing the recruitment of electrically-coupled cells, an effect that is reverted when cells receive coincident excitation. Furthermore, a new functional consequence of coincidence detection is highlighted: the regulation of the latency of action potential generation by coupled cells. Paired recordings performed here are a proof of principle of the phenomenon. The magnitude of the effects is expected to be larger when a network of several coupled cells is simultaneously activated.

The probability that BCs generate action potentials in response to glutamatergic stimulation is higher in Cx36−/− mice lacking ESs among BCs and their latency is shorter, suggesting that “adding” ESs to the network decreases the cellular response to incoming excitatory inputs, due to current leaking out of cells through ESs. At the steady state, the leakage of current through ESs has been shown to make a major contribution to the input conductance of electrically coupled cells (Alcami and Marty, 2013). The results of the present article confirm that the current leaking through ESs is also relevant at (chemical glutamatergic) synaptic time scales, affecting integrative properties of BCs. EPSC amplitude does not differ in WT and Cx36−/− mice BCs, and no obvious compensation in leak channels seems to counteract the absence of current leakage through ESs in the hyperpolarizing range (Alcami and Marty, 2013). However, it cannot be ruled out that compensatory mechanisms in Cx36−/− may have changed the way BCs respond to glutamatergic inputs in Cx36−/− mice. Compensatory mechanisms have indeed been described in networks connected by Cx36-mediated ESs (De Zeeuw et al., 2003).

In line with the interpretation according to which the leak of current through ESs present in WT but not Cx36−/− mice explains the increased latency and decreased probability of action potentials in WT mice, a long-lasting simultaneous suprathreshold depolarization of a coupled cell induced an increase in firing rate (Figure 1) and a decrease in action potential latency (Figure 2), likely by reducing the leak through the ES between the two cells. Furthermore, action potential probability strongly increased and their latency showed a sub-millisecond decrease in response to brief simultaneous near-threshold current injections in an electrically-coupled cell, only when cells were electrically coupled (Figures 3, 4). These short injections mimic the short-conductance change that typically occurs at chemical excitatory synapses. All these results concur to suggest that owing to the presence of ESs, coupled BCs behave as coincidence detectors (Rela and Szczupak, 2004; Hjorth et al., 2009). They are less responsive to non-coincident stimuli, but they are activated more strongly and faster when their coupled partners are simultaneously depolarized. Coincidence detection abilities of electrically-coupled cells are dynamic (they can show short-term depression in response to trains of stimuli) and frequency-dependent, these properties being likely related to the properties of signal propagation through ESs and to the interaction of coupling with the intrinsic properties of BCs. The influence of subcellular location of ESs, likely to impact coincidence detection abilities, should be further investigated.

### Sequence Detection by Electrically-Coupled Cells

In addition, individual BC firing is maximally facilitated when a cell is sequentially activated shortly after a coupled cell. Thus, coupled cells are sequence detectors in addition to coincidence detectors. As a consequence, a BC activated shortly after another coupled BC is expected to inhibit postsynaptic targets (here Purkinje cells) with an even higher probability and shorter latency than when both BCs are coincidentally stimulated. How general is the ability of coupled cells to enhance their firing in response to a sequential activation? The time window for the facilitation of action potential firing by the excitation of coupled cells studied here extends for tens of milliseconds, mirroring the kinetics of average postsynaptic potentials induced by presynaptic near-threshold stimulations. These result from a combination of both postsynaptic spikelets in reponse to presynaptic action potentials and of slower-decaying coupling potentials evoked by presynaptic subthreshold stimulations (Figure 6B). A finer exploration of short-duration stimulation intervals of coupled cells should be performed in order to determine the most effective interval to enhance their firing responses.

Electrically-coupled neocortical interneurons were reported to increase their firing probability when stimulated 1 ms but not 5 ms after one another at depolarized membrane potentials, suggesting that the facilitation of firing in response to sequences can occur in narrower time-windows (Galarreta and Hestrin, 2001b). Action potentials reliably evoked by strong suprathreshold stimuli in a Golgi cell inhibit coupled cells activated sequentially at delays lasting 10 ms or longer due to the large hyperpolarizing component of spikelets (Vervaeke et al., 2010). In contrast, spikelets in BCs do not show strong hyperpolarizing phases, and spikelet-induced depolarizations are amplified by the interaction with intrinsic currents (Mann-Metzer and Yarom, 1999). Since intrinsic properties determine the waveform of action potentials, spikelets and coupling potentials, they are likely to determine the time-window for coincidence and sequence detection. Likewise, the value of the membrane potential of coupled cells receiving coincident inputs is expected to modulate the time-window for enhanced firing. It would be interesting to explore in detail the modulation of the time-window for enhanced interneuron recruitment as a function of the membrane potential and the intrinsic properties of coupled cells. Faster membrane potential kinetics in adult animals may further reduce the time window for coincidence detection.

Note that in Figures 3, 4, 6, in contrast with some previous studies (e.g., Vervaeke et al., 2010), stimuli are near-threshold in both cells, evoking firing of both coupled cells in a probabilistic manner. They generate subthreshold events or action potentials with a given probability and variable latency in each stimulated cell. Combined, they generate the extended time-window for enhanced firing observed here. Future work should dissect the mechanisms and the relative contribution of subthreshold and suprathreshold responses in facilitating firing in coupled cells (van Welie et al., 2016).

Coincident or near-coincident excitation is relevant in the cerebellar cortex given the synchrony and the geometry of excitatory afferents and the effectiveness of excitatory inputs to trigger BC action potentials. In other coupled networks, stimuli rarely occur in perfect coincidence, in regular trains or in isolation from other inputs, but instead, they are likely to occur at variable delays in the context of an intense synaptic bombardment *in vivo* (Destexhe et al., 2013). The impact of coincidence and sequence detection abilities of ESs in networks subject to numerous inputs with a complex temporal structure still remains to be explored.

### Recruitment of BCs and Cerebellar Computation

In the cerebellar cortex, BCs are activated in spatial clusters by groups of simultaneously active neighboring axons (Eccles et al., 1967; Cohen and Yarom, 1998; Cramer et al., 2013). Since electrical coupling incidence decreases as a function of inter-somatic distance (Rieubland et al., 2014), the strength and speed of BC recruitment are expected to be maximally increased when all coupled neighbors receive inputs with high temporal correlation at the center of an excited region. By contrast, cells situated at its edge are likely to be less active and more slowly recruited due to a smaller number of simultaneously-depolarized coupled neighbors, and they may be further hyperpolarized by the electrotonic spread of lateral inhibition. The position of BCs in space relative to the region targeted by active presynaptic axons at a given time and the size of activation will determine how many coupled neighbors are coincidentally activated, and thereby how strongly and how fast BCs are activated. Therefore, the activation of a large enough network of BCs is expected to generate action potentials with variable latencies and probabilities, respectively increasing and decreasing from the center to the edge of the activated network. In other words, the latency and the probability of interneuron recruitment in response to the activation of a given excitatory synapse are not fixed, but they instead depend on ES connectivity, on the geometry and temporal pattern of excitation in the network.

A Purkinje cell receiving both an excitatory input and a fast and strong inhibitory input from interneurons coincidentally activated (at the center of an activated region) may be dominated by inhibition and may respond with a silence in action potential firing. However, the same Purkinje cell receiving longer-latency inhibitory inputs from interneurons activated this time at the border of an activated region of the cerebellar cortex may respond with a classical feed-forward inhibitory pattern, excitation not being prevented in this last scenario from reaching action potential threshold by inhibition. In this manner, the geometry of excitation of the cerebellar cortex combined with the relative position of interneurons and their connectivity through ESs may determine whether Purkinje cells respond with action potentials or, on the contrary, with pauses in action potentials to an activation of the cerebellar cortex. In response to sensory stimulation *in vivo*, Purkinje cells receive a strong and fast inhibition from molecular layer interneurons, which often prevents excitatory events from triggering action potentials (Chu et al., 2012). Coincident excitation of electrically-coupled interneurons may be at play to achieve such a fast and efficient inhibition, preventing Purkinje cells from firing despite receiving a direct excitatory input.

This result can be generalized to other feed forward inhibitory circuits, where postsynaptic principal cells may be dominated by a fast and strong inhibition when presynaptic electrically-coupled interneurons receive excitatory inputs with a high temporal correlation. The submillisecond decrease in BC action potential latency by the coincident activation of coupled cells, combined with the higher probability that coincident inputs trigger action potentials may constitute a mechanism allowing interneurons to generate a highly-efficient inhibition of postsynaptic cells.

### General Implications

The results show that the probability and latency of action potentials generated by BCs, and thereby the impact of the inhibition that they generate in postsynaptic targets, carries the signature of their order of activation. The impact of electrically-coupled interneurons onto their postsynaptic targets is therefore variable and conditioned by the detection of the temporal structure of excitatory inputs onto BCs by ESs. It is generally assumed that the high efficiency of inhibition is exquisitely shaped and constrained by morphology, chemical connectivity and by the properties of chemical synaptic transmission between interneurons and principal cells (Hu et al., 2014). The present results suggest that additional players, ESs, influence the efficiency of inhibition by controlling interneuron recruitment as a function of the spatio-temporal pattern of stimulation of the interneuron network.

The knowledge of how neurons are connected is necessary to infer the function of nervous systems (Bargmann and Marder, 2013). The present article shows that the impact of inhibition and the properties of microcircuits involving electrically-coupled neurons cannot be understood without the knowledge of a connectome that includes ESs. Indeed, the connectivity through ESs will determine the properties of interneuron recruitment and thereby the most effective presynaptic temporal pattern of activity to ultimately inhibit a postsynaptic cell. Unfortunately current large-scale connectomics and modeling approaches focus on one of the two modalities of synaptic transmission, chemical synapses, overlooking ESs (Markram et al., 2015; Mikula and Denk, 2015).

Electrical synapses have been shown to be plastic, among others by regulating their conductance (Pereda et al., 1998; Zsiros and Maccaferri, 2008; Haas et al., 2011). Since coincidence detection abilities are stronger for larger conductances (Figure 3D), changes in the strength of electrical connections between interneurons are expected to reconfigure the flow of current in the network, thereby regulating its coincidence detection abilities and in that manner, the efficiency and speed of recruitment of inhibitory circuits.

## Author Contributions

PA designed experiments, performed experiments, analyzed the data and wrote the article.

## Conflict of Interest Statement

The author declares that the research was conducted in the absence of any commercial or financial relationships that could be construed as a potential conflict of interest.

## Footnotes

^1^http://www.physiologie.uni-freiburg.de/research-groups/neural-networks

^2^www.neuromatic.thinkrandom.com
